# Impact of Unannounced Standardized Patient Audit and Feedback on Care, Documentation, and Costs: an Experiment and Claims Analysis

**DOI:** 10.1007/s11606-020-05965-1

**Published:** 2020-07-07

**Authors:** Alan Schwartz, Steven Peskin, Alan Spiro, Saul J. Weiner

**Affiliations:** 1 Institute for Practice and Provider Performance Improvement, Inc., 3712 N. Broadway #460, Chicago, IL 60613 USA; 2 Horizon Blue Cross Blue Shield of New Jersey, Newark, NJ NJ USA; 3 Rutgers Robert Wood Johnson Medical School, New Brunswick, NJ USA

**Keywords:** unannounced standardized patients, quality improvement, health services research, cost of care, directly observed care

## Abstract

**Background:**

Meaningful variations in physician performance are not always discernible from the medical record.

**Objective:**

We used unannounced standardized patients to measure and provide feedback on care quality and fidelity of documentation, and examined downstream effects on reimbursement claims.

**Design:**

Static group pre-post comparison study conducted between 2017 and 2019.

**Setting:**

Fourteen New Jersey primary care practice groups (22 practices) enrolled in Horizon BCBS’s value-based program received the intervention. For claims analyses, we identified 14 additional comparison practice groups matched on county, practice size, and claims activity.

**Participants:**

Fifty-nine of 64 providers volunteered to participate.

**Intervention:**

Unannounced standardized patients (USPs) made 217 visits portraying patients with 1–2 focal conditions (diabetes, depression, back pain, smoking, or preventive cancer screening). After two baseline visits to a provider, we delivered feedback and conducted two follow-up visits.

**Measurements:**

USP-completed checklists of guideline-based provider care behaviors, visit audio recordings, and provider notes were used to measure behaviors performed and documentation errors pre- and post-feedback. We also compared changes in 3-month office-based claims by actual patients between the intervention and comparison practice groups before and after feedback.

**Results:**

Expected clinical behaviors increased from 46% to 56% (OR = 1.53, 95% CI 1.29–1.83, *p* < 0.0001), with significant improvements in smoking cessation, back pain, and depression screening. Providers were less likely to document unperformed tasks after (16%) than before feedback (18%; OR = 0.74, 95% CI 0.62 to 0.90, *p* = 0.002). Actual claim costs increased significantly less in the study than comparison group for diabetes and depression but significantly more for smoking cessation, cancer screening, and low back pain.

**Limitations:**

Self-selection of participating practices and lack of access to prescription claims.

**Conclusion:**

Direct observation of care identifies hidden deficits in practice and documentation, and with feedback can improve both, with concomitant effects on costs.

**Electronic supplementary material:**

The online version of this article (10.1007/s11606-020-05965-1) contains supplementary material, which is available to authorized users.

## INTRODUCTION

Health care delivery is rarely systematically directly observed.^1, 2^ Clinicians vary in effective practice based on how well they listen and ask key questions of patients; these variations are not captured using current quality measures.^3, 4^

In past research, we used “unannounced standardized patients” (USPs) to measure clinician performance during direct observation. USPs are actors trained to present to clinicians incognito as patients, portraying standardized scripts that facilitate controlled comparisons among practices and providers.

Using USPs, we have studied the impact of physician inattention to patient psychosocial issues relevant to care planning, termed “patient contextual factors.”^5–7^ In a study of 400 USP visits, internists who overlooked clues that patients’ clinical problems were related to contextual factors were more likely to order unnecessary tests and therapies, with a median excess cost of $231 per visit.^8^ USP projects have also found discrepancies between the medical record and what actually occurred during the visit. Others employing USPs have reported nearly a third of recorded physical exam maneuvers do not occur.^9, 10^ These lapses are not discoverable without direct observation.

We have advocated for the widespread implementation of USPs as a performance assessment and improvement strategy.^1, 11, 12^ USPs unmask widespread and serious deficits in care quality that are otherwise invisible and that, if remediated, would reduce overuse and misuse of medical services.^13, 14^ One approach to remediation (“audit and feedback”) is to give providers feedback based on USP assessments of their performance.^15^ The goal is to positively modify the way they care for real patients.

The purpose of this study was to use USP visits and feedback to assess and improve the quality of primary care delivered and documented, and to measure the impact on cost of care in actual patients.

## METHODS

### Impact on Quality of Care

#### Setting and Cases

We partnered with Horizon BCBS of New Jersey to define target areas aligned with Horizon’s value-based program, which incentivizes practices based on care metrics related to the target areas. Four USP case scripts were designed around diabetes and idiopathic low back pain, with opportunities for providers to address medication adherence (diabetes), opioid use (low back pain), depression screening (all cases, with positive screens in two cases), smoking cessation (two cases), and reluctance to engage in recommended cancer screenings (three cases). Measures were of ordinarily unmeasurable physician behaviors, e.g., whether they followed CDC guidelines for promoting smoking cessation or simply told the patient they should “quit.” Appendix 1 shows the conditions and expected behaviors associated with each case script.

USPs were recruited from the standardized patient and professional theater communities in the local area. They received eight hours of in-person training prior to their first visit. Script development and training methods were similar to those used in past research.^7^

We recruited New Jersey primary care providers (physicians and nurse practitioners) in practices enrolled in Horizon’s value-based program. Providers were told that they would receive visits from four USPs during the 18 months of the project (without remuneration), and provided informed consent. Physician participants could receive MOC part IV credit from the American Board of Internal Medicine or American Board of Family Medicine. Practices identified a staff confederate to assist in scheduling visits and ensuring that USPs would be seen (despite lack of insurance), to transmit copies of provider notes to the researchers following USP visits, and to “white out” electronic medical records of USP visits to prevent their inclusion in billing or practice quality reporting metrics. Horizon staff did not have access to identified provider- or practice-level data.

#### Design

Providers were visited by four USPs, each playing one of the case scripts, in a counterbalanced order, and divided into two phases of two visits each; providers were randomized to case orders. For each visit, the provider was emailed after obtaining their note to inform them they had seen a USP and to ask whether they had believed they were seeing a real patient or a USP. Following the first two visits to all providers within a practice, each provider was given reports of their performance and their practice’s aggregate performance, and participated in a coaching phone call with a physician investigator (SW) with input from a quality improvement specialist from the American College of Physicians. This process was repeated after the two following visits. Reports included, for each case, visualizations of the proportion of times the practice or individual provider performed each expected care behavior (as compared with all practices and providers in the study), visualizations of document fidelity (how often visit tasks were performed or not vs. documented or not), and, in the practice reports, CAHPS clinician and group survey measures and narrative comments from the USPs, with the provider not identified.

#### Measures

USPs completed a checklist of guideline-based diagnostic and treatment provider behaviors. USPs also covertly audio recorded the visits. The practice confederate provided the provider’s encounter note and any intake paperwork used for the visit. We coded visit recordings and provider notes for case-specific performance indicators (e.g., was depression screening correctly performed (audio) and did physician document the task (note)?) We considered information on the intake paperwork as if it had been provided by the patient in the encounter, and we considered a depression screen to have been performed if the screen appeared in the paperwork. Our fidelity measure included four categories for each indicator: heard on audio and documented in note (correct), heard on audio but not documented (undocumented), not heard on audio, not documented in note (unperformed), and not heard on audio but documented (fictitious).

#### Data Analyses

To examine the association between visit time (pre- vs. post-feedback) and quality-of-care behaviors, we fitted a mixed-effects logistic regression model to the USP checklist items, with fixed effects of visit time and SP case, and random effects of practice, provider, and checklist item to control for clustering of items in cases in providers in practices. We also examined whether physician suspicion they had seen a USP was associated with performance. To examine the association between visit time and documentation fidelity, we fitted a similar mixed-effects multinominal logistic regression model. We sought a sample size of 60 providers overall based on a priori 80% power to detect an improvement from a baseline rate of 40% to a post-intervention rate of 65% in a given performance indicator, based on previous studies.

#### Role of the Funding Source

Support for this project was provided by a grant from the Robert Wood Johnson Foundation to the American College of Physicians and the Institute for Practice and Provider Performance Improvement, Inc. The funding agreement ensured the independence of the investigators in the design, conduct, and analysis of the study.

### Impact on Cost Patterns

#### Comparison Practices

For billing purposes, practices are associated with taxpayer identification numbers (TINs); several co-owned practices could share one TIN. Prior to claims data collection, we matched each study practice TIN to a comparison practice TIN in the same county and also enrolled in the Horizon value-based program, using propensity score matching^16^ based on practice size (members enrolled) and activity (two different 5-level ordinal measures of aggregate claims billed to Horizon) in 2016. A total of 117 TINs were available for matching; the matched group was well balanced on size (SMD = − 0.02, *p* = 0.96) and better balanced on activity measures than the population (SMD = 0.17 and − 0.20).

Horizon provided costs of actual claims paid for inpatient, outpatient, and professional services (primary or specialty care) in the three months prior to the project (incurred October–December 2017 and paid October 2017–March 2018), the three months 9 months later (after feedback, incurred July–September 2018 and paid July–December 2018), and the three months 12 months later (incurred October–December 2018 and paid October 2018–March 2019), in all visits by (actual) patients attributed to study and comparison practices who had received at least one service associated with a focal condition (diabetes, opioid use, depression, back pain, smoking, or cancer screening) during at least one visit. We considered a visit in the per-visit claims data to be associated with a condition if it included any claim with any ICD-10 code associated with the condition (see Appendix 2 for ICD-10 codes by condition), but included claims for all care that occurred in the same visit. Claims data did not include prescription claims, capitation claims, or patient-paid portions of charges (i.e., co-pays and deductibles) and were identified at the TIN level. For diabetes and low back pain, Horizon also provided patient-level costs in each period using Horizon’s internal attribution algorithm, which aggregates only those claims related to the condition, and computes total disease-related cost of the claims for members receiving care for the disease.

Claim “costs” represent costs to Horizon, not societal costs, but provide a numeraire that does not differ between intervention and control practice groups. Not all claims had an associated cost to Horizon; we refer to those that do as “positive claims.”

#### Data Analyses

We fitted two-part mixed-model regressions for each focal condition to per-visit claims costs: we modeled the probability of having a positive claim with a logistic model and the cost of positive claims with a gamma model. In each model, we include a fixed effect of practice type (study vs. comparison), a fixed effect of time period (pre-feedback, 9 months later, and 12 months later), the interaction between practice type and time period, and a random effect of practice TIN to account for clustering of claims in TINs. We examined the difference-in-difference of predicted per-visit costs between comparison and study practices at the 9-month and 12-month periods (each compared with the pre-feedback cost as a baseline). We fitted similar two-part mixed regressions to patient-attributed costs for diabetes and for low back pain. We hypothesized significant practice type × time interactions, with the direction depending on the nature of the focal condition.

**Table 1:** Raw Proportions and Adjusted Odds Ratios for Quality of Care Pre- and Post-feedback

Checklist item	Pre-feedback proportion	Post-feedback proportion	Adjusted odds ratio (95% CI)	*p* value
Overall (all areas)	46%	56%	1.53 (1.29–1.83)	< 0.001
Identifies reason for diabetes medication nonadherence	59%	60%	1.04 (0.46–2.37)	0.92
Addresses diabetes medication nonadherence	51%	50%	0.97 (0.45–2.05)	0.93
Manages low back pain without opioids	74%	84%	2.17 (1.01–4.59)	0.048
Identifies resistance to cancer screening	39%	34%	0.71 (0.43–1.20)	0.20
Addresses resistance to cancer screening	27%	29%	0.84 (0.53–2.19)	0.84
Identifies depression	52%	70%	3.03 (1.43–6.45)	0.004
Addresses depression when identified	14%	24%	1.93 (0.79–4.71)	0.15
Provides evidence-based smoking cessation guidance	24%	58%	9.35 (4.68–18.7)	< 0.001

Note: Odds ratio for overall performance is adjusted for case and clustering of responses by item, provider, and practice. Odds ratios for individual items are adjusted for clustering of cases in providers

For privacy reasons, Horizon’s per-visit claims could not identify whether different visits were by the same patients, so we treated each visit as an independent patient. The patient-level cost data does not require this assumption. We analyzed claims in raw dollars in the period they were incurred (thus, main effects of time include any changes in reimbursement rates over the study duration). One author (AS) conducted analyses using R 3.6^17^ with the lme4^18^ and mgcv^19^ packages and Julia 1.1.1^20^ with the MixedModels^21^ package. The project was approved by Advarra IRB.

## RESULTS

### Quality of Care Measured by USPs

Twenty-two study practices (representing 14 TINs) agreed to participate; eight were solo practices, 10 were group practices with 2–3 providers, and 4 were group practices with more providers (range 4–14). Of the 64 total providers at these practices, 59 agreed to participate.

USPs made 217 visits in total between May 2017 and March 2019. Four practices had only pre-feedback visits (because the practice closed, participating providers died or moved, or actors were unable to schedule post-feedback visits in a timely fashion); the remaining 18 had pre-feedback and post-feedback visits. Of the providers, 48 received all four visits, 4 received 3 visits, 6 received 2 pre-feedback visits only, and one received a single pre-feedback visit. Providers suspected they had seen a USP in 19 visits, believed they had seen a real patient in 73 visits, and did not reply to the email in the remaining visits.

#### Care Provided

Providers performed expected care based on USP checklists in 46% of items in pre-feedback visits and 56% in post-feedback visits (OR = 1.53, 95% CI 1.29–1.83, *p* < 0.001). Significant improvement areas included smoking cessation, managing chronic low back pain without opioids, and depression screening (Table 1). Belief that they had seen a USP vs. a real patient vs. not responding was not associated with provider performance (*χ*^2^(2) = 1.09, *p* = 0.58).

#### Documentation Fidelity

**Figure 1:**
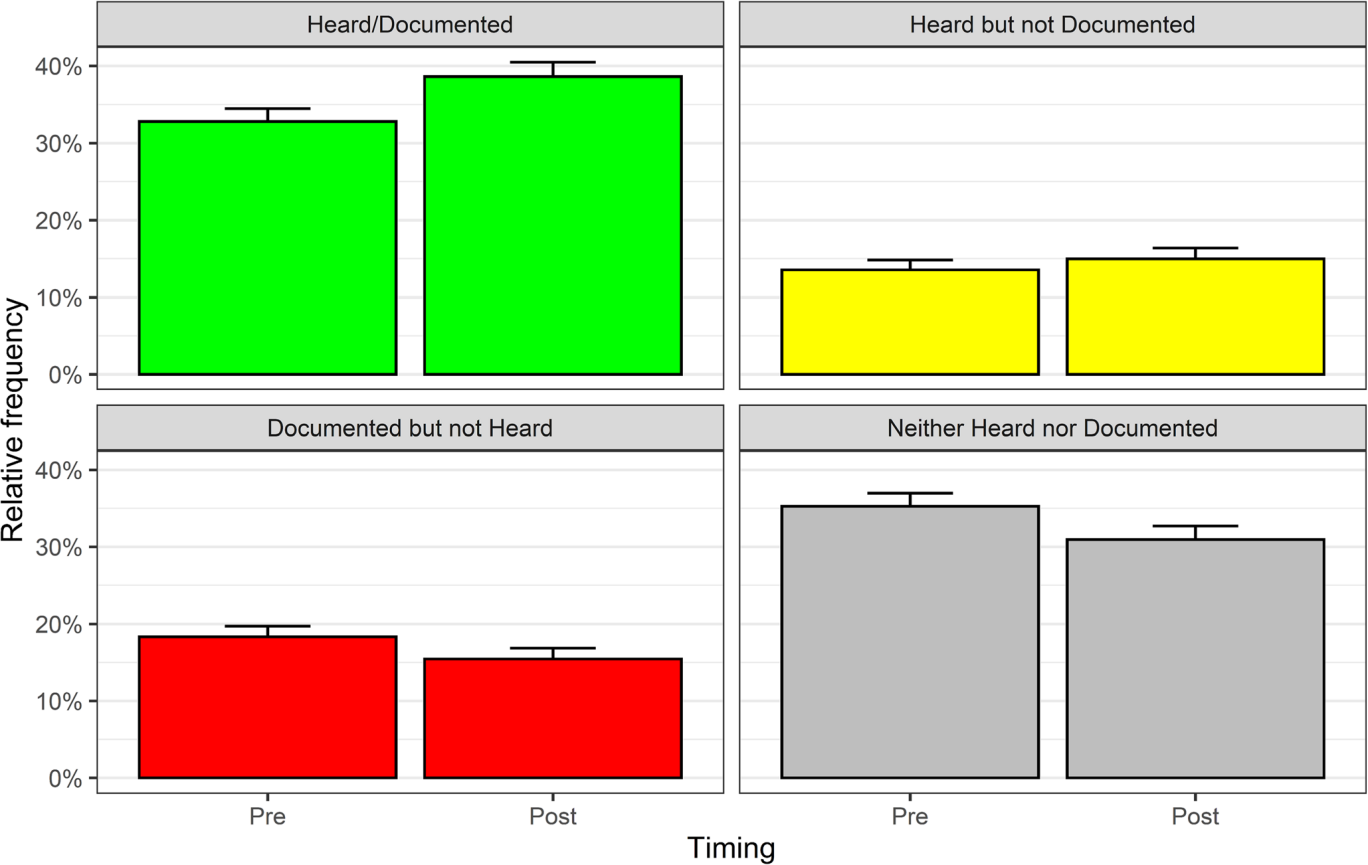
**Observed frequency of targeted behaviors recorded by USPs (“Heard”) vs. entered in the medical record (“Documented”) in pre- and post-feedback visits. Error bars indicate the upper limits of the 95% confidence intervals around the proportions.**

Post-feedback visits had lower rates of unperformed behaviors (OR = 0.67, 95% CI 0.57 to 0.78, *p* < 0.001) and of fictitious documentation (OR = 0.74, 95% CI 0.62 to 0.90, *p* = 0.002) than pre-feedback visits. Unperformed behaviors increased for cancer screening and decreased for depression and smoking cessation. Rates of undocumented behaviors did not differ overall by post- vs. pre-feedback timing (OR = 0.89, 95% CI 0.74 to 1.06, *p* = 0.19), but were higher for medical adherence in diabetes and lower for review of systems. Figure 1 presents overall unadjusted rates and Table 2 adjusted odds ratios for groups of items.

**Table 2:** Changes in Documentation Fidelity from Pre- to Post-feedback overall and for groups of items

Checklist item	Unperformed behavior adjusted odds ratio (95% CI)	*p* value	Fictitious documentation adjusted odds ratio (95% CI)	*p* value	Undocumented behavior adjusted odds ratio (95% CI)	*p* value
Overall (all items)	0.67 (0.57–0.78)	< 0.001	0.74 (0.62–0.90)	0.002	0.89 (0.74–1.06)	0.19
Diabetes	1.03 (0.69–1.54)	0.90	1.31 (0.87–1.98)	0.20	2.94 (1.33–6.46)	0.008
Low back pain	0.57 (0.25–1.32)	0.19	0.62 (0.33–1.16)	0.14	N/A	
Cancer screening	1.90 (1.15–3.14)	0.01	1.04 (0.63–1.71)	0.88	2.02 (0.42–9.79)	0.38
Depression	0.17 (0.11–0.28)	< 0.001	1.06 (0.54–2.09)	0.86	1.38 (0.61–3.14)	0.44
Smoking	0.11 (0.06–0.22)	< 0.001	0.84 (0.46–1.54)	0.57	0.54 (0.22–1.31)	0.17
Review of systems	0.87 (0.67–1.13)	0.29	0.94 (0.67–1.33)	0.74	0.62 (0.49 – 0.79)	<0.001

Notes: Odds ratios are relative to correct documentation of behaviors performed and heard on the visit recording, and adjusted for case, item, and provider clustering. For low back pain, there were not a sufficient number of undocumented behaviors to model the odds ratio

### Cost for Care in Actual Patients

Overall, per-visit claims were more frequent and considerably more expensive in post-feedback than pre-feedback periods, regardless of study group. Per-visit claims related to study focal conditions totaled $17,104,906 (235,644 claims) pre-feedback (Oct–Dec 2017), $48,787,157 (316,116 claims) nine months after feedback (Jul–Sep 2018), and $48,112,140 (307,277 claims) twelve months after feedback (Oct–Dec 2018).

#### Overall Patterns in Claims

The rate of positive claims for focal conditions was 86.8% overall and did not differ significantly between study and comparison practice groups at any time period for any focal condition except cancer screening. The number of patients with $0 per-patient-attributable costs similarly did not differ between groups at any time period.

**Figure 2:**
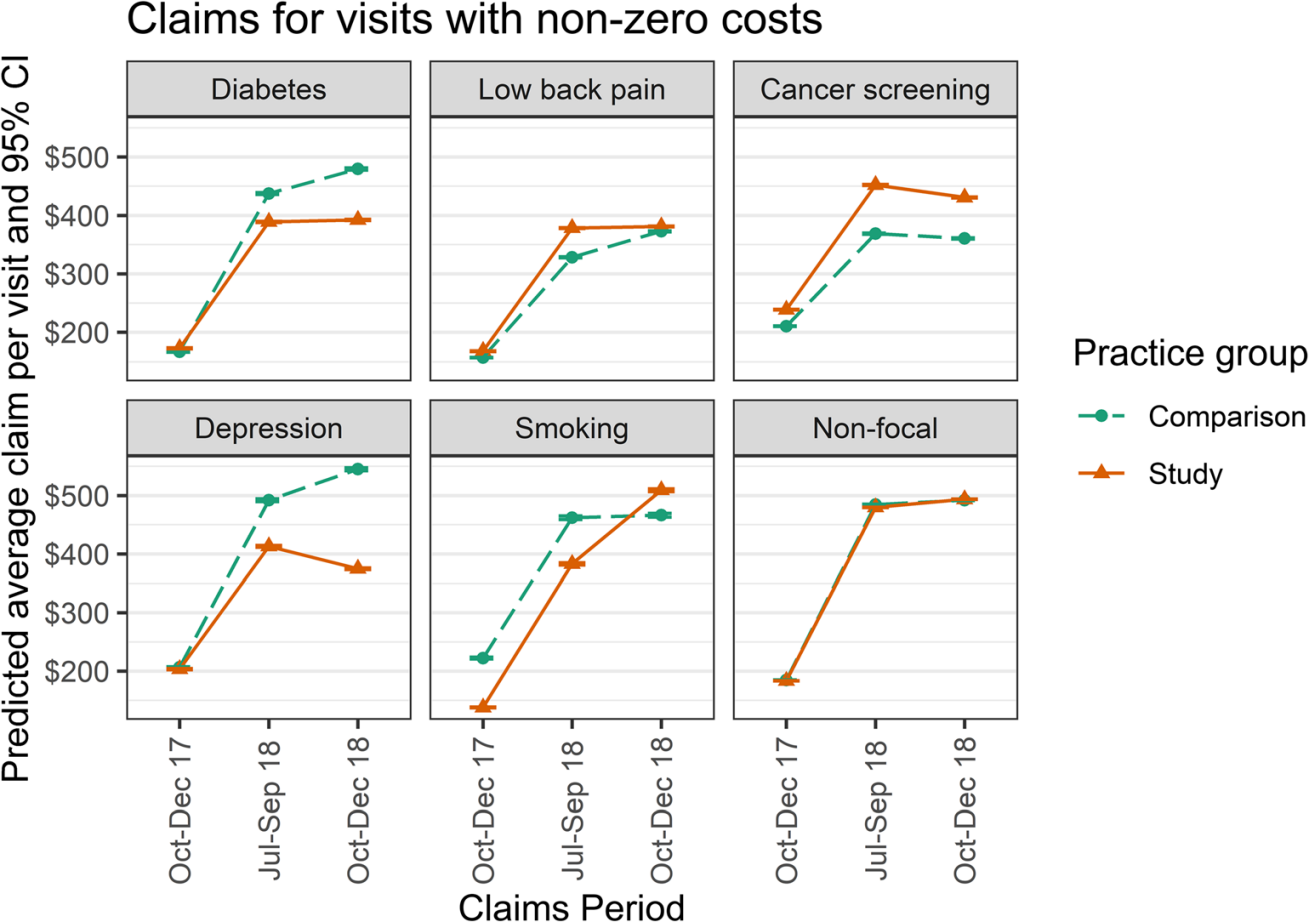
**Predicted average per-visit claims by group, time period, and focal condition.**

Table 3 presents gamma regression coefficients and standard errors for per-visit office-based claims for each of the focal conditions, as well as per-visit difference-in-difference costs (pre-post change in the study TINs vs. the comparison TINs); Table 4 presents the same for per-patient-attributable costs for diabetes and low back pain. Figure 2 shows the predicted average claim per visit by groups and time periods for each condition, and for all other claims (“non-focal”).

**Table 3:** Gamma Regression Coefficients (Per-Visit Data for Visits with Positive Claims) and Difference-in-Differences of Claims Costs

	Diabetes	Low back pain	Cancer screening	Depression	Smoking cessation
Predicted effect on office costs	Improved medication adherence in study group leads to lower office costs associated with DM	Reduced reliance on opioids in study group leads to higher costs associated with office-delivered modalities	Increased rate of screening in study group leads to higher costs	No prediction (increased screening leads to some increased costs for office-based therapy and some decreased costs for office-based management of sequelae)	Improved attention to tobacco leads to higher office costs associated with cessation interventions (e.g., motivational interviewing)
Number of visits	58,479	112,287	100,973	30,662	17,933
Regression coefficients (standard error)					
Intercept	5.01 (0.08)	5.03 (0.07)	5.33 (0.08)	5.34 (0.09)	5.33 (0.16)
Type (study vs. intervention)	0.01 (0.11)	0.06 (0.10)	0.06 (0.11)	− 0.26 (0.13)	− 0.09 (0.22)
Time (9 months vs. baseline)	0.86 (0.02)*	0.70 (0.01)*	0.49 (0.01)*	0.78 (0.02)*	0.54 (0.03)*
Time (12 months vs. baseline)	0.95 (0.02)*	0.84 (0.01)*	0.48 (0.01)*	0.89 (0.02)*	0.57 (0.03)*
Type (study) × time (9 months)	− 0.11 (0.02)*	0.07 (0.01)*	0.08 (0.02)*	− 0.12 (0.03)*	0.15 (0.04)*
Type (study) × time (12 months)	− 0.20 (0.02)*	−0.06 (0.01)*	0.02 (0.02)	− 0.34 (0.03)*	0.29 (0.04)*
Difference-in-differences					
Difference-in-difference in claims costs (baseline vs. 9 months)	− $54.08	$39.39	$30.17	− $75.92	$3.51
Difference-in-difference in claims costs (baseline vs. 12 months)	− $92.78	− $2.25	$16.30	− $167.22	$127.25

Note: ^*^p < 0.05; difference-in-difference costs are per-visit and incorporate both expected differences in the number and average amount of positive-dollar claims; negative difference-in-differences favor the study group; positive difference-in-differences favor the comparison group

In all conditions, there was no significant difference in claim dollars at baseline between study and comparison group practices, and average claim dollars significantly increased over time periods in both groups. Among non-focal conditions, claims also did not differ significantly by group.

**Table 4:** Gamma Regression Coefficients (Patient-Level Data) and Difference-in-Differences in Per-Patient Costs

	Diabetes	Low back pain
Prediction	Improved medication adherence in study group leads to lower office costs associated with DM	Reduced reliance on opioids in study group leads to higher costs associated with office-delivered modalities
Number of patients	8777	7069
Regression coefficients		
Intercept	7.3 (0.13)	7.3 (0.14)
Type (study vs. intervention)	− 0.08 (0.18)	− 0.28 (0.19)
Time (9 months vs. baseline)	− 0.10 (0.04)^*^	− 0.31 (0.05)^*^
Time (12 months vs. baseline)	0.05 (0.04)	− 0.11 (0.05)^*^
Type (study) × time (9 months)	− 0.36 (0.05)^*^	0.53 (0.06)^*^
Type (study) × time (12 months)	− 0.57 (0.06)^*^	0.51 (0.06)*
Difference-in-differences		
Difference-in-difference in claims costs (baseline vs. 9 months)	− $500.80	$781.47
Difference-in-difference in claims costs (baseline vs. 12 months)	− $841.75	$867.15

Note: *p<0.05; Negative difference-in-differences favor the study group; positive difference-in-differences favor the comparison group

#### Diabetes

Study practices showed significantly lower rates of increase in office-based diabetes claims than the comparison practices; this was reflected in both per-visit and per-patient costs.

#### Low Back Pain

Study practices showed significantly higher rates of increase in office-based low back pain claims than the comparison practices at 9 months (per-visit and per-patient) and 12 months (per-patient only).

#### Cancer Screening

In cancer screening, study and comparison groups were equally likely to have positive claims at the pre-feedback time period (adjusted OR = 0.85, 95% CI 0.72–1.02), but the study group was significantly more likely to have positive claims than the comparison group at both the 9-month and 12-month periods (adjusted OR = 1.36, 95% CI 1.18–1.56 in each period compared with baseline for study vs. comparison group). Study practices also showed significantly higher rates of increase in office-based screening claims than the comparison practices at 9 (but not 12) months. Combining the greater frequency and amount of positive claims over time in the study vs. comparison group, study practices showed significantly higher rates of increase in office-based cancer screening claims than comparison practices.

#### Depression

Study practices showed significantly lower rates of increase in office-based depression claims than the comparison practices.

#### Smoking Cessation

Study practices showed significantly higher rates of increase in office-based smoking cessation claims than the comparison practices.

## DISCUSSION

In this study, we employed unannounced standardized patients (USPs) to directly observe physician behaviors at managing common ambulatory conditions and compared findings to what they documented in the medical record. We discovered previously undetectable deficits in clinician performance and documentation, and after providing feedback and working with practices to develop quality improvement strategies, quality of care and documentation improved.

We also observed significant and expected changes to claims costs in the care of real patients among practices who participated in the intervention relative to the comparison group. Specifically, actual claims costs increased less among real patients with diabetes and depression, where physicians adopted recommended behaviors that reduced costs when caring for USPs, and claims costs increased more among real patients receiving care for smoking addiction, low back pain, and preventive cancer care, where physicians adopted recommended behaviors that increased costs.

We did not have access to prescription claims. Although the observed changes in costs in the care of real patients were consistent with what we observed in the care of the USPs, we cannot confirm that they were due to the same desired increases or decreases in prescribing. For instance, although physical therapy claims went up as expected, we cannot confirm that opioid prescribing went down. In one area, cancer prevention, however, the improvements we saw in the care of USPs matched those exhibited in the care of real patients.

Two observations from the claims analysis were unexpected. Claims increased substantially overall between the pre- and post-feedback time periods. The increase may reflect changes in reimbursement policy or shifts in membership over the insurance year; our comparison group helps us control for this. For low back pain, the per-visit claims costs increased more for the study than comparison groups at 9 months, but not 12 months, while the per-patient costs increased more for the study than comparison groups at both periods. This may reflect a decreasing impact of our intervention over time.

Because practices elected to participate in the project, differences seen in the study practices may not represent the effect that would be observed in all practices. The matched (a priori) comparison group and use of common time periods mitigates this limitation, but if the study practices differ substantially from the typical practice, our matching could have led to similarly atypical comparison practices.

USPs provide a penetrating assessment of clinician performance because they assess actual clinician behaviors rather than what is recorded in the medical record. They enable highly personalized feedback that providers can utilize to improve their care and potentially enhance value-based care. In our project, the marginal cost of an additional two USP visits to a provider (not including overhead) with feedback and coaching was approximately $700, a fraction of the potential cost savings associated with improved patient care for conditions where higher quality reduces office-based care (e.g., diabetes medication adherence). For conditions where improving quality increases short-term costs (e.g., preventive care), payers with sufficiently long-term horizons may realize cost savings from future improved health. In short, direct observation of care identifies deficits in practice and documentation, and can improve both, sometimes with predictable concomitant cost savings.

## Electronic supplementary material

ESM 1 (DOCX 33 kb)

## References

[1] **Weiner SJ**, **Schwartz A**. Directly observed care: can unannounced standardized patients address a gap in performance measurement?*. J Gen Intern Med* 2014. 10.1007/s11606-014-2860-7 PMC4099461 24756945

[2] Stange KC, Zyzanski SJ, Jaen CR (1998). Illuminating the 'black box'. A description of 4454 patient visits to 138 family physicians. J Fam Pract..

[3] National Center for Quality Assurance. http://www.ncqa.org/HEDISQualityMeasurement/PerformanceMeasurement.aspx, 2013.

[4] Agency for Healthcare Research and Quality. CAHPS clinician & group surveys. Available at: https://cahps.ahrq.gov/Surveys-Guidance/CG/index.html. Last accessed May 10, 2014.

[5] Weiner SJ (2004). Contextualizing medical decisions to individualize care: lessons from the qualitative sciences. J Gen Intern Med..

[6] **Weiner SJ**. Contextual error. In: Kattan M, ed. Encyclopedia of medical decision making: SAGE; 2009:198-202.

[7] Weiner SJ, Schwartz A, Weaver F (2010). Contextual errors and failures in individualizing patient care: a multicenter study. Ann Intern Med.

[8] **Schwartz A**, **Weiner SJ**, **Weaver F**, et al. Uncharted territory: measuring costs of diagnostic errors outside the medical record. *BMJ Qual Saf.* 2012. 10.1136/bmjqs-2012-000832 22773889

[9] Dresselhaus TR, Luck J, Peabody JW (2002). The ethical problem of false positives: a prospective evaluation of physician reporting in the medical record. J Med Ethics.

[10] Dresselhaus TR, Peabody JW, Lee M, Wang MM, Luck J (2000). Measuring compliance with preventive care guidelines: standardized patients, clinical vignettes, and the medical record. J Gen Intern Med.

[11] **Weiner SJ**, **Schwartz A**, **Cyrus K**, **Binns-Calvey A**, **Weaver FM**, **Sharma G**, **Yudkowsky R** Unannounced standardize patient assessment of the roter interaction analysis system: the challenge of measuring patient-centered communication. *Journal of General Internal Medicine.* 2012:In Press. 10.1007/s11606-012-2221-3 PMC3614126 22990681

[12] **Weiner SJ, Schwartz A**. *Listening for what matters: avoiding contextual errors in health care.* New York, NY: Oxford University Press; 2016.

[13] **Weiner SJ, Schwartz A**, **Sharma G**, **Binns-Calvey A**, **Ashley N**, **Kelly B**, **Weaver FM**. Patient collected audio for performance assessment of the clinical encounter. *Jt Comm J Qual Patient Saf* 2015;42(6):273-278. 10.1016/s1553-7250(15)41037-2 25990893

[14] Schwartz A, Weiner SJ, Harris IB, Binns-Calvey A (2010). An educational intervention for contextualizing patient care and medical students' abilities to probe for contextual issues in simulated patients. JAMA..

[15] **Ivers N**, **Jamtvedt G**, **Flottorp S**, **Young JM**, **Odgaard-Jensen J**, **French SD**, **O'Brien MA**, **Johansen M**, **Grimshaw J**, **Oxman AD**. Audit and feedback: effects on professional practice and healthcare outcomes. Cochrane Database of Systematic Reviews 2012, Issue 6. Art. No.: CD000259. DOI: 10.1002/14651858.CD000259.pub3. . 10.1002/14651858.CD000259.pub3 PMC11338587 22696318

[16] **Ho D**, **Imai K**, **King G**, **Stuart EA**. MatchIt: nonparametric preprocessing for parametric causal inference. *2011.* 2011;42(8):28.

[17] *R: A Language and Environment for Statistical Computing* [computer program]. Vienna, Austria2013.

[18] **Bates D**, **Maechler M**, **Bolker B**, **Walker S**. lme4: linear mixed-effects models using Eigen and S4. 2013.

[19] Wood S (2017). *Generalized additive models: an introduction with R*.

[20] Bezanson J, Edelman A, Karpinski S, Shah VB (2017). Julia: A fresh approach to numerical computing. SIAM review..

[21] **Bates D.** MixedModels.jl: mixed-effects models in Julia. 2018; https://github.com/dmbates/MixedModels.jl.

