# Supplementary material for: Impact of Unannounced Standardized Patient Audit and Feedback on Care, Documentation, and Costs: an Experiment and Claims Analysis
Source: J Gen Intern Med. 2020 Jul 7;36(1):27–34. doi: 10.1007/s11606-020-05965-1 (PMC7859004; doi:10.1007/s11606-020-05965-1)
Supplement: Supplementary file 1 — (DOCX 33 kb) [file 11606_2020_5965_MOESM1_ESM.docx]

# Appendices

## Appendix 1: USP cases and expected provider behaviors associated with each portrayed condition

| Case | Conditions | Expected provider behaviors* |
| --- | --- | --- |
| A | Diabetes medication adherence | Provider identifies reasons patient is not taking diabetes medication and addresses them in care plan |
|  | Depression | Provider correctly screens or reviews results of a screen for depression utilizing an evidence based screening instrument and, addresses barriers to receiving care for depression when screens positive and meets diagnostic criteria. |
|  | Cervical cancer screening reluctance | Providers notes patients’ reluctance to engage in screening, identifies reasons, and addresses them in care plan. |
| B | Diabetes medication adherence | As above |
|  | Smoking cessation | Evidence based recommendations include 3 components: Advising smokers and tobacco users to quit, discussing cessation medications, discussing cessation strategies. |
|  | Colon cancer screening reluctance | As above (See cervical cancer screening reluctance) |
| C | Low back pain (opioid requested) | Providers appropriately manages chronic non-cancer pain in a patient requesting opioids. |
|  | Depression | As above |
|  | Smoking cessation | As above |
| D | Low back pain (opioid requested) | As above |
|  | Mammography reluctance | As above (See cervical cancer screening reluctance) |

*Across all four USP

## Appendix 2: ICD-10 codes used to associate visits with conditions

| Condition | Diagnostic code prefixes | Procedure codes |
| --- | --- | --- |
| Depression | F31  F32  F33  GZ6 | 80332  80333  80334  80335  80336  80338  90791  90792  90801  90802  90820  90825  90830  90835  90841  90842  90843  90844  90845  90849  90853  90857  1220F  3700F  3725F  4060F  4062F  4064F  4065F  4306F  4320F |
| Diabetes | E08  E09  E10  E11  E13  L97  B35.1 | 148  11055  11056  11057  11719  11720  11721  92002  92002  92004  92004  92012  92012  92014  92014  92225  92225  92226  92226  97802  97803  97804  0403T  0488T  2022F  2022F-8P  2024F  2024F-8P  2026F  2026F-8P  2028F  3044F  3045F  3046F  G0127 |
| Low back pain | M46  M47  M48  M50  M51  M53  M54  F11 | 97000  97001  97002  97030  97050  97100  97200  97780  97781  97810  97811  97813  97814  98940  98941  98942  98943 |
| Cancer screening | Z01  R87  Z11  Z12  Z13 | 44388  44389  44390  44391  44392  44393  44401  44402  44403  44404  44405  44406  44407  44408  45330  45331  45333  45334  45336  45337  45339  45341  45342  45345  45355  45360  45365  45367  45368  45369  45370  45371  45372  45378  45379  45380  45381  45382  45383  45384  45385  45386  45387  45388  45389  45390  45391  45392  45393  45398  74261  74262  74263  74263  76092  76093  76094  76095  76097  76640  76641  76642  76645  77032  77055  77056  77057  77058  77059  77061  77062  77063  82270  82271  82272  82274  84443  84800  87620  87621  87622  87623  87624  87625  88141  0066T  0067T  0500T  G0101 |
| Smoking cessation | HZ37ZZZ  Z71  F17  Z72 | 99406  99406  99407  99407  0002F  0003F  0004F  1001F  1031F  1034F  4000F |
